# Hepatitis C Virus-Infected Apoptotic Hepatocytes Program Macrophages and Hepatic Stellate Cells for Liver Inflammation and Fibrosis Development: Role of Ethanol as a Second Hit

**DOI:** 10.3390/biom8040113

**Published:** 2018-10-13

**Authors:** Murali Ganesan, Larisa Y. Poluektova, Chijioke Enweluzo, Kusum K. Kharbanda, Natalia A. Osna

**Affiliations:** 1 Research Service, Veterans Affairs Nebraska-Western Iowa Health Care System, Omaha, NE 68105, USA; murali.ganesan@unmc.edu (M.G.); kkharbanda@unmc.edu (K.K.K.); 2 Department of Internal Medicine, University of Nebraska Medical Center, Omaha, NE 68198, USA; cj.enweluzo@unmc.edu; 3 Department of Pharmacology and Experimental Neuroscience, University of Nebraska Medical Center, Omaha, NE 68198, USA; lpoluekt@unmc.edu

**Keywords:** acetaldehyde, HCV, apoptotic bodies, liver inflammation, fibrosis, ethanol

## Abstract

Hepatocyte apoptosis is a crucially important mechanism for liver disease pathogenesis, and the engulfment of apoptotic bodies (AB) by non-parenchymal cells serves as a leading mechanism of inflammation and fibrosis progression. Previously, we have shown that hepatitis C virus (HCV) and alcohol metabolites induce massive apoptosis in hepatocytes and the spread of HCV-infection to the neighboring uninfected cells. Here, we hypothesize that the capturing of AB by non-parenchymal cells, macrophages and hepatic stellate cells (HSC) changes their phenotype to promote inflammation and fibrosis. In this regard, we generated AB from Huh7.5^CYP2E1^ (RLW) cells also treated with an acetaldehyde-generating system (AGS) and incubated them with human monocyte-derived macrophages (MDMs) and HSC (LX2 cells). Activation of inflammasomes and pro-fibrotic markers has been tested by RT-PCR and linked to HCV expression and AGS-induced lipid peroxidation in RLW cells. After exposure to AB we observed activation of inflammasomes in MDMs, with a higher effect of AB HCV+, further enhanced by incubation of MDMs with ethanol. In HSC, activation of inflammasomes was modest; however, HCV and AGS exposure induced pro-fibrotic changes. We conclude that HCV as well as lipid peroxidation-adducted proteins packaged in AB may serve as a vehicle for delivery of parenchymal cell cargo to non-parenchymal cells to activate inflammasomes and pro-fibrotic genes and promote liver inflammation and fibrosis.

## 1. Introduction

Hepatitis C virus (HCV)-infection causes chronic hepatitis accompanied with fibrosis and frequent progression to liver cirrhosis. The incidence of hepatocellular carcinoma (HCC) in HCV^+^ cirrhotic patients is about 3.5-fold higher that in cirrhosis of non-viral etiologies (alcoholic liver diseases (ALD) and non-alcoholic fatty liver disease (NAFLD)) [1,2]. It is also known that second hits, such as alcohol intake or co-infection with human immunodeficiency virus (HIV) significantly increase HCC risk development [3,4].

Hepatocyte apoptosis is a crucially important mechanism for liver disease pathogenesis, and the engulfment of apoptotic bodies (AB) by non-parenchymal cells serves as a leading mechanism of inflammation and fibrosis progression [5]. Activation of immune response for elimination of virus-permissive cells is a crucial element of anti-viral protection. Liver macrophages (Mph) play a significant role in this process. They express the receptors for pathogen-associated molecular patterns (PAMPs), as well as recognize and phagocytose pathogens to destroy them. However, in the process of virus elimination, activated by PAMPs, Mphs secrete pro-inflammatory cytokines, which induce liver inflammation to occur in the HCV-infected liver [6]. Similarly, engulfment of apoptotic hepatocytes expressing non-structural HCV proteins by hepatic stellate cells (HSC) may activate liver fibrosis [7]. Both short-term liver inflammation and fibrosis are beneficial for liver regeneration if it occurs in the frame of acute (cyclic) processes accompanied by the clearance of pathogen. In contrary, in chronic hepatotropic infections like Hepatitis B virus (HBV) and as shown recently, HIV (especially, triggered by exposure to alcohol), when hepatocyte death become a constant event, multiple attempts to remove apoptotic hepatocytes by non-parenchymal cells cause persistent liver inflammation and fibrosis. This might be the case for hepatitis C since it results in chronic infection in 70–80% cases [8].

Previously, we have shown that the alcohol metabolite acetaldehyde promotes apoptosis in HCV-infected hepatocytes, and these AB not only contain HCV, but also spread the infection to non-infected neighboring hepatocytes [9]. Furthermore, in the same study, we demonstrated that the prevention of apoptosis by the treatment with pan-caspase inhibitor increased the level of HCV RNA in HCV-replicating hepatocytes exposed to an acetaldehyde generating system (AGS), indicating that the death of HCV-infected hepatocytes and subsequent dissemination of HCV infection can be enhanced by alcohol metabolism [9]. Here, we hypothesize that the engulfment of HCV-containing hepatic AB by both Mph and HSC program the phenotype of non-parenchymal cells and promote pro-inflammatory (for Mph) and pro-fibrotic (for HSC) changes in these cells. To this end, we mimicked the apoptotic effects of acetaldehyde by exposure of HCV^+^ and HCV^−^ hepatocytes to ultraviolet (UV) light and then incubated these AB with human Mph (monocyte-derived macrophages (MDM)) and HSC (LX2 cells) to measure the induction of inflammasomes and the expression of pro-inflammatory cytokines and pro-fibrotic markers.

## 2. Materials and Methods

### 2.1. Reagents and Media

High glucose Dulbecco’s Modified Eagle Medium (DMEM) and fetal bovine serum were purchased from Invitrogen (Carlsbad, CA, USA). Trizol was sourced from Life Technologies, Primer probes and RT-PCR reagents such as TaqMan Universal Master Mix II, with UNG and High Capacity cDNA Reverse Transcription Kit were from Applied Biosystems by Thermo Fisher Scientific Ashveville, NC, USA). Cytochrome P450 E1 (CYP2E1) was from EMD Millipore (Temecula, CA, USA) and Anti- alcohol dehydrogenase (ADH) was a gift from Dr. Michael Felder (University of South Carolina). Other reagents, all of analytical grade quality, were from Sigma (St. Louis, MO, USA).

### 2.2. Huh7.5-CYP Cells and Treatments

Huh7.5^CYP2E1^ (RLW) cells (Huh 7.5 cells stably transfected with CYP2E1, [10,11]) were infected or not with HCV (JFH1 virus, MOI 0.1), for 96 h. In some experiments, RLW cells were also treated with an AGS which is a mixture of 50 mM ethanol, recombinant ADH and nicotinamide adenine dinucleotide (NAD) that releases physiologically relevant amounts of acetaldehyde generated in the process of alcohol metabolism [10,11]. Specifically, the cells were infected with HCV for 48 h and then exposed to AGS for another 48 h.

### 2.3. Primary Human Macrophages

Monocytes were obtained from healthy donor blood elutriation. For Mph (here, monocyte-derived Mph, MDM) differentiation, human monocytes were cultured for 0–8 days in DMEM culture media containing human serum and macrophage colony-stimulating factor.

### 2.4. Hepatic Stellate Cells

As the source of HSC, we used the commercially available human cell line, LX2 (EMD Millipore, cat SCC064) grown based on instructions from the manufacturer.

### 2.5. Apoptotic Bodies Generation and Characterization of Apoptotic Cells

Apoptotic bodies were generated from either uninfected or HCV-infected RLW cells and characterized as described before [9]. Briefly, apoptosis was induced by UV light (0–100 mJ/cm^2^, 140 s). After 24 h, the floating apoptotic bodies were collected from 100mm plates by centrifugation at 1500 rpm for 5 min and resuspended in DMEM. As demonstrated by us before [9], the percentage of apoptotic RLW cells was determined using the annexin-V/propidium iodide (PI) apoptosis detection kit by flow cytometry (BD Biosciences, San Diego, CA, USA). In addition, Hoechst 33342 staining of normal and apoptotic cells has been performed for visualization of apoptotic morphological features, such as DNA condensation, fragmentation, and shrinkage in UV light-exposed cells.

### 2.6. Macrophage and HSC Treatment with AB

After differentiation of MDMs for 8 days, on day 9, an equal number of AB from HCV-infected RLW cells or HCV-infected RLW cells pretreated with AGS were added to MDMs for 2, 4, 6, and 18 h (MDM:AB ratio was 1:3). AGS-treated or untreated HCV noninfected cells used for AB generation served as a negative control for the effects of virus/viral proteins on inflammasome markers, cytokine and fibrotic markers based on mRNA induction in MDMs and HSCs measured by RT-PCR, as described above. For most parameters, maximal effects of AB on gene activation in MDMs were observed as early as at 2h of AB incubation with the cells.

### 2.7. RNA Isolation and Real-Time PCR

Total RNA was isolated from cells using Trizol Reagent (Thermo Fisher Scientific, Asheville, NC, USA). A 2-step procedure was applied, in which 200 ng RNA was reverse-transcribed to cDNA using the high capacity reverse transcription kit. Then the cDNA was amplified using TaqMan Universal Master Mix-II with fluorescent-labeled primers (TaqMan gene expression systems). After incubation in a Model 7500 qRT-PCR thermal cycler (Applied Bioscience, Foster City, CA, USA), relative quantity of each RNA transcript was calculated by its threshold cycle (Ct) after subtracting that of the reference cDNA (*GAPDH*). Data were expressed as the quantity of transcript (RQ). HCV infection was confirmed by the measurement of HCV RNA levels. The relative HCV RNA expression level in infected cells was quantified using the primers and probes for this consensus sequence, which were designed using PrimerExpress Software v2.0 (Applied Biosystems): 5’UTRF GACCGGGTCCTTTCTTGGAT; 5’UTRR CCAACACTACTCGGCTAGCAGTCT; probe FAM-ATTTGGGCGTGCCCCCGC-NFQ.

### 2.8. Immunoblotting (Western Blot)

Cell lysates prepared in 0.5 M Ethylenediaminetetraacetic acid (EDTA), 2 M Tris, 20 mM Na3VO4, 200 mM Na4P2O7, 100 mM phenylmethylsulfonyl fluoride (PMSF), 1 M NaF, 20% Triton X-100, and aprotinin, pH 7 were separated and subjected to immunoblotting technique as previously described [11]. Blots were developed using Odyssey infrared imaging system (Li-Cor Bioscience, Lincoln, NE, USA), and the protein band was quantified using Li-Cor Bioscience software.

### 2.9. Statistical Analyses

Data from at least three duplicate independent experiments are expressed as mean values ± standard error. Comparisons among multiple groups were determined by one-way ANOVA, using a Tukey post-hoc test. For comparisons between two groups, we used the Student’s *t*-test. A probability value of 0.05 or less was considered significant.

## 3. Results

Previously, we have shown that acetaldehyde continuously released by an AGS induces apoptosis in HCV-infected RLW cells recapitulating the pro-apoptotic effects of ethanol on HCV-exposed primary human hepatocytes [12]. These AB spread HCV infection to intact neighboring hepatocytes [9]. Here, we will test the effects of AB from HCV-infected and uninfected cells on inflammasome activation in MDMs. Since large amounts of AB were required for these experiments, they were generated by exposure of HCV^+^ and HCV^−^ RLW cells to UV light. The MDMs were then exposed to these AB as stated in Materials and Methods.

### 3.1. Induction of Inflammasome, Pro-Inflammatory Cytokines and TGFβ in MDMs Exposed to AB from HCV^+^ and HCV^−^ RLW Cells

Production of inflammatory cytokines is regulated by inflammasome activation via NOD-like receptors (NLRs), which are pattern-recognition receptors triggered by pathogens and non-viral danger signals. Activation of inflammasomes requires expression of the inflammasome receptor, NLRP3 that recognizes HCV as an RNA virus, serving as the priming signal to initiate the transcription of pre-interleukin (IL)-1β and pre-IL-18. Inflammasome induction causes activation of caspase-1 to induce the maturation of IL-1β and IL-18.

**Figure 1:**
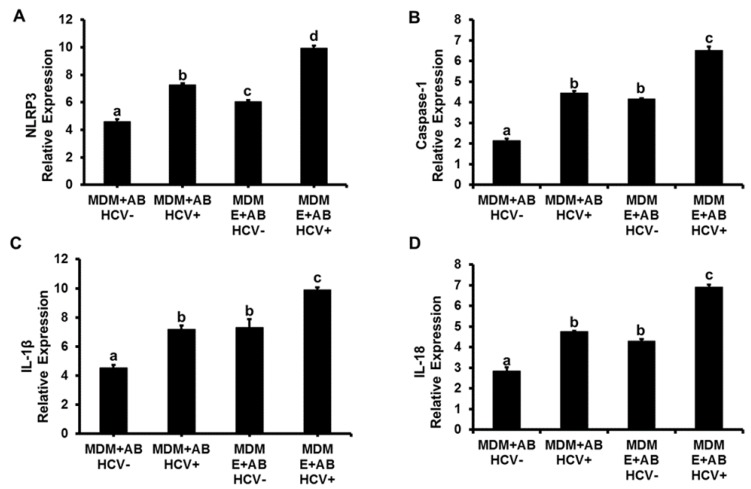

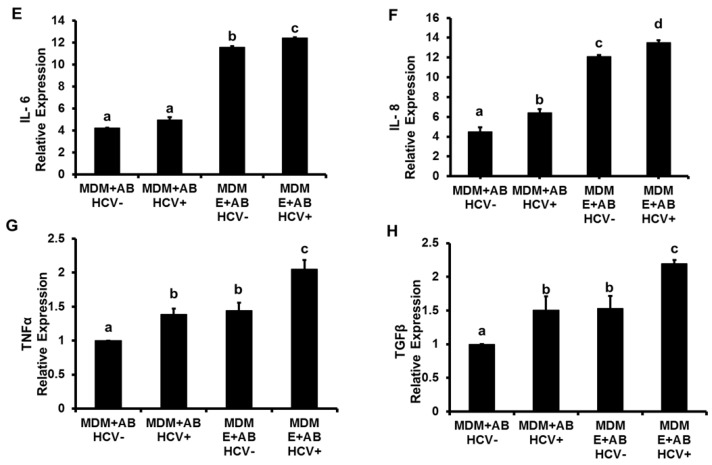
Activation of inflammasome in monocyte-derived macrophages (MDMs) by hepatitis C virus (HCV)-infected apoptotic bodies (AB) engulfment: Huh7.5^CYP2E1^ (RLW) cells were either non-infected or infected with HCV-JFH1 virus for four days then AB were collected after ultraviolet (UV) light exposure as mentioned in the Methods section and incubated with non-treated or 48 h-pre-treated with 50 mM ethanol (E) MDMs for 2 h. mRNA expression of (**A**) *NLRP3*, (**B**) *Caspase-1*, (**C**) *IL-1β* and (**D**) *IL-18* were measured by RT-PCR analysis. MDMs not exposed to AB were used as a control. *GAPDH* was used as an internal control for all RT-PCR experiments. Induction of cytokine expression by AB engulfment in MDMs: mRNA expression of cytokines (**E**) *IL-6*, (**F**) *IL-8*, (**G**) *TNFα* and (**H**) *TGFβ* were measured by RT-PCR analysis. Data are from three independent experiments presented as means ± standard error (SE). Bars marked with the same letter are not significantly different from each other; bars with different letters are significantly different (*p* ≤ 0.05).

As shown in Figure 1A, expression of NLRP3 in MDMs was upregulated by engulfment of AB generated from HCV-infected RLW (AB_HCV+RLW_) cells compared with the engulfment of AB from HCV-negative RLW (AB_HCV−RLW_) cells (*p* < 0.05). When MDMs were pre-treated with 50 mM ethanol (E) two days prior to incubation with AB, the effects of AB_HCV+RLW_ on *NLRP3* expression became even more evident. The same pattern was observed for *caspase-1* (Figure 1B), *IL-1β* and *IL-18* genes (Figure 1C,D), which was further confirmed by the three-fold increase in IL-1β and five-fold increase in IL-18 cytokine production in cell supernatants, as quantified by ELISA (DuoSet ELISA, R&D Systems, Minneapolis, MN, USA) 48 h after exposure of MDMs to AB_HCV+-RLW_. We also found that AB_HCV+RLW_ triggered an increase in i*nterleukin* (*IL)-6*, *IL-8*, *tumor necrosis factor α (TNF)α* and *transforming growth factor β* (*TGFβ*) gene expression. The levels of these cytokine mRNAs were enhanced up to two-fold when AB were incubated with ethanol-pre-treated MDMs (Figure 1E–H).

### 3.2. Effects of Ethanol Exposure to MDMs on AB-Induced Inflammasome Activation

**Figure 2:**
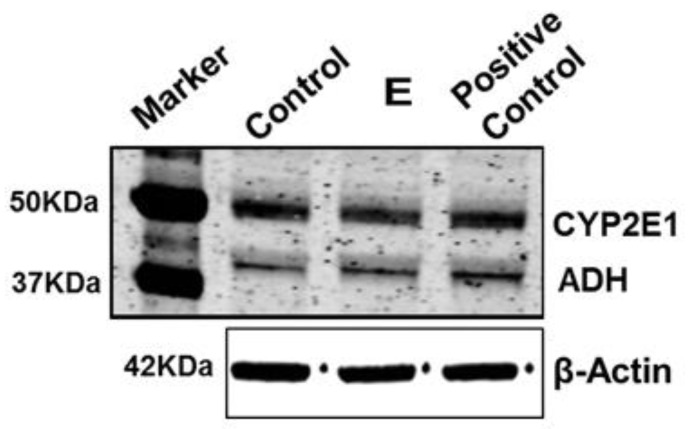
Effect of ethanol exposure to MDM on expression of ethanol-metabolizing enzymes: After 8–10 days differentiation of monocytes into MDM, cells were treated with 50 mM ethanol for 48 h. Ethanol (E) treatment was done every 24 h. Expressions of ethanol-metabolizing enzymes, ADH and CYP2E1, were measured by immunoblotting (IB). β-actin was used as an internal control. Each lane was loaded with 20 µg of protein. Primary human hepatocytes were used as the positive control.

Since ethanol exposure to MDMs significantly increased the activation of inflammasomes, TGFβ and pro-inflammatory cytokine genes in response to engulfment of AB_HCV+RLW_, we wondered whether ethanol-metabolizing enzymes, CYP2E1 and ADH are expressed in MDMs and whether incubation of cells with ethanol affects their expression levels. MDMs were exposed to 50 mM ethanol for 48 h and then CYP2E1 and ADH were quantified in cell lysates by immunoblotting (IB); CYP2E1-and ADH-expressing hepatocytes were used as a positive control. Figure 2 demonstrates that both CYP2E1 and ADH are presented in MDMs and thus, MDMs metabolize ethanol via these enzymes. However, ethanol exposure does not induce CYP2E1 stabilization as previously observed in hepatocytes [13,14].

### 3.3. Effects of AGSExposure to RLW Cells on AB-Triggered Activation of Inflammasomes in MDMs

**Figure 3:**
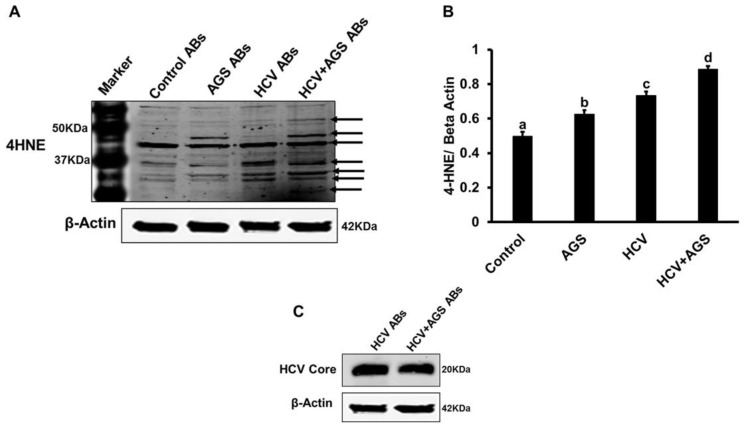
Effects of the acetaldehyde generating system (AGS) on lipid peroxidation adduct formation in RLW cells: RLW cells were either non-infected or infected with HCV-JFH-1 virus for 2 days, then treated with AGS for 48 h. AB collected after UV light exposure were intensively washed and lysed for immunoblotting. (**A**,**C**) 4-hydroxynonenal (4-HNE) adducts and HCV core protein were measured by immunoblotting. (**B**) Quantification of 4-HNE immunoblotting. Equal amounts (20 µg) of protein were loaded in each lane. β-actin was used as an internal control. Data are from three independent experiments presented as means ± SE. Bars marked with the same letter are not significantly different from each other; bars with different letters are significantly different (*p* ≤ 0.05).

Next, we asked whether the effects of AB on inflammasome induction depend on expression of HCV or lipid peroxidation markers in hepatocytes. In some experiments, AB were made from HCV-infected and uninfected hepatocytes also exposed to AGS (AB_HCV+AGS+_). These treatments of RLW cells induce lipid peroxidation, which causes 4-hydroxynonenal (4-HNE) or malondialdehyde (MDA)-protein adduct formation. The adducts may potentially affect induction of pro-inflammatory markers in MDMs incubated with adductexpressing AB and are presented in HCV-infected AGS-treated RLW cells [9]. To prove that AB_HCV+AGS+_ contain 4-HNE-and MDA-adducted proteins, IB was performed using adduct-specific antibodies (Alpha Diagnostics, Int, San Antonio, TX, USA). We also measured the level of HCV core protein in AB_HCV+RLW_. As shown in Figure 3A–C, while AGS treatment in AB_HCV+RLW_ attenuated HCV core protein expression in apoptotic RLW cells, there was no difference in MDA expression in AB_HCV+AGS+_ and AB_HCV−AGS+_. However, 4-HNE-adducts were present in AB generated from the cells exposed to HCV or AGS, with the highest expression in those isolated from doubly treated cells. These AB from AGS-treated cells differentially expressed increases in a few yet non-identified 4-HNE-adducted proteins, which were not observed in the absence of AGS (Figure 3A).

**Figure 4:**
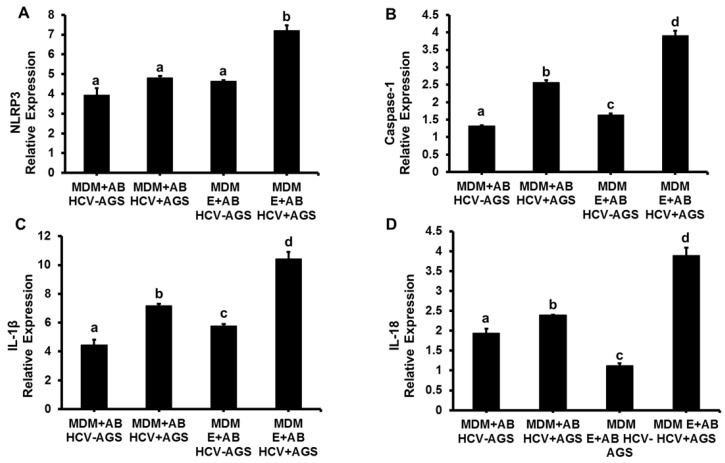
Effect of AGS on AB-triggered activation of inflammasome in MDMs: RLW cells were either non-infected or infected with HCV-JFH-1 virus for two days then treated with AGS for 48 h. AB were collected after UV light exposure and incubated with MDM (pre-treated or not with E) for 2 h. Then mRNA expression of (**A**) *NLRP3*, (**B**) *caspase-1*, (**C**) *IL-1β* and (**D**) *IL-18* were measured by RT- PCR analysis. MDMs not exposed to AB were used as a control. Data are from three independent experiments presented as means ± SE. Bars marked with the same letter are not significantly different from each other; bars with different letters are significantly different (*p* ≤ 0.05).

The pattern of inflammasome activation in MDMs by AB_HCV+_ and AB_HCV−_ from AGS-untreated and treated cells was the similar, but the magnitude of response to AB_HCV+AGS+_ induced increase in NLRP3, caspase 1, IL-1β and IL-18 mRNAs expression was lower when AB were generated from AGS-treated RLW cells compared with the effects of AB generated from AGS-non-treated cells (Figure 4A–D vs. Figure 1A–C, compare the scales in these figures). Consistent with the pattern of inflammasome activation in MDMs by AB_HCV+AGS−_, the pretreatment of MDMs with ethanol potentiated the inflammasome induction by AB_HCV+AGS+_ exposure to MDMs on (Figure 4A–D).

### 3.4. Induction of Inflammasome and Pro-Fibrotic Response in Hepatic Stellate Cells (HSC) Exposed to AB from HCV^+^ and HCV^−^ RLW Cells

Like macrophages, HSC can also engulf AB and become activated. Here, we studied whether incubation of HSC with AB from HCV^+^ and HCV^−^ RLW cells in the presence or absence of AGS induced inflammasome activation and pro-fibrotic response in HSC. As a source of human HSC, we used the LX-2 cell line.

#### 3.4.1. Pro-Inflammatory HSC Activation by AB

**Figure 5:**
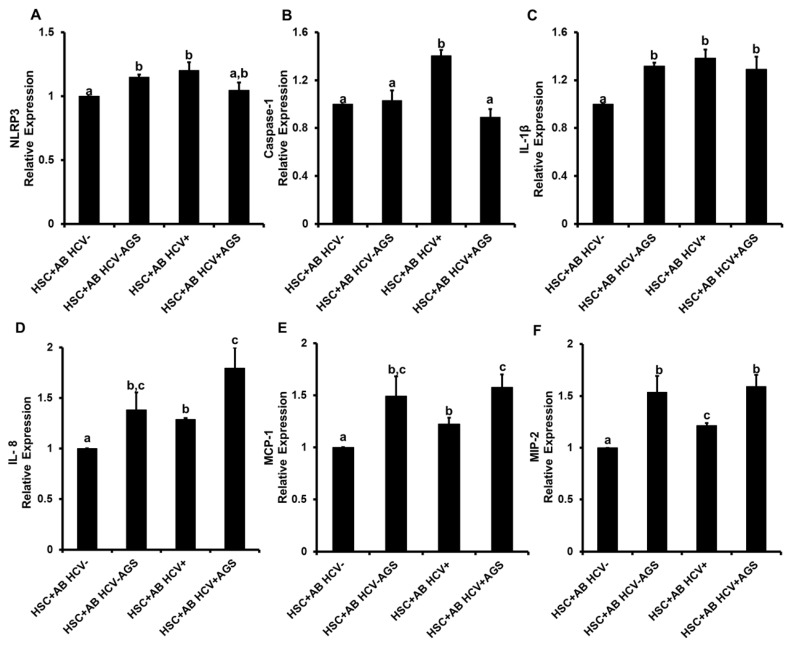
AB-mediated effects of HCV and AGS on pro-inflammatory cytokines in hepatic stellate cells (HSC) (LX2 cells): RLW cells were either non-infected or infected with HCV-JFH-1 virus for two days then treated or not with AGS for 48 h. AB were collected after UV light exposure and incubated with LX2 cells for 2 h. mRNA expression of (**A**) *NLRP3*, (**B**) *Caspase-1*, (**C**) *IL-1β,* (**D**) *IL-8*, (**E**) monocyte chemotactic protein 1 (*MCP-1*) and (**F**) macrophage inflammatory protein 2 (*MIP-2*) were measured by RT PCR analysis. Data are from three independent experiments presented as means ± SE. Bars marked with the same letter are not significantly different from each other; bars with different letters are significantly different (*p* ≤ 0.05).

AB were generated from HCV+ and HCV- RLW cells either exposed or not to AGS and incubated with HSC as indicated above. To characterize inflammasome activation, mRNA expression of *NLRP3*, *caspase- 1* and *IL-1β* were measured. We observed a modest activation (~25%) of *NLRP3* by both AB_HCV+_ and AB_AGS+_ (Figure 5A), 40% induction of *caspase-1* by AB _HCV_ (Figure 5B), as well as *IL-1β* activation (about 30%) by AB_HCV+_ and AB_AGS+_ (Figure 5C). Expression of *IL-8* in HSC was also activated in response to AB_HCV+_ and AB_AGS+_, *p* < 0.05) (Figure 5D). In addition to pro-inflammatory cytokine activation, we also tested the effects of AB on chemokine (monocyte chemotactic protein 1 (MCP-1 and macrophage inflammatory protein 2 (MIP-2)) induction in HSC, which is critical for promoting infiltration of the inflammatory cells to the liver, thereby contributing to fibrosis development. AB_HCV+_ modestly activated both MCP-1 and MIP-2 mRNAs expression in HSC, but AB_AGS_+ induced ~50% increase in these two chemokines (Figure 5E,F). Pre-treatment of HSC with ethanol (50 mM, 48 h) did not affect inflammatory response of HSC to AB (not shown).

#### 3.4.2. Pro-Fibrotic HSC Activation by AB

**Figure 6:**
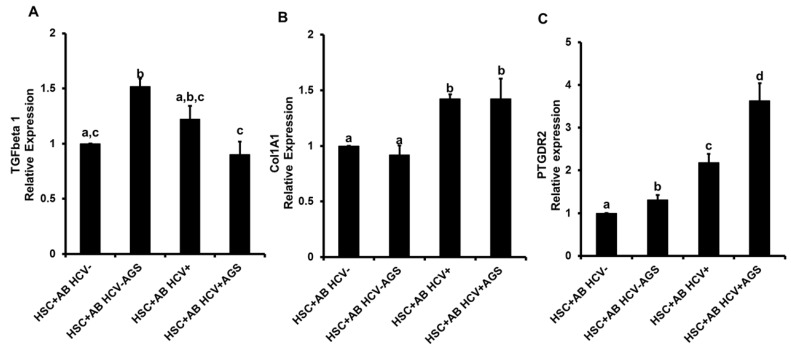
AB-mediated effects of HCV and AGS on pro-fibrotic markers in HSC (LX2 cells): RLW cells were either non-infected or infected with HCV-JFH-1 virus for two days, then treated or not with AGS for 48 h. AB were collected after UV light exposure and incubated with LX2 cells for 2 h. Then mRNA expression of (**A**) *TGFβ1,* (**B**) *collagen 1 A1 (Col1A1)* and (**C**) *prostaglandin D receptor 2 (PTGDR2)* were measured by RT PCR analysis. Data are from three independent experiments presented as means ± SE. Bars marked with the same letter are not significantly different from each other; bars with different letters are significantly different (*p* ≤ 0.05).

Here, after exposure of HSC to AB, we quantified the expression of pro-fibrotic markers, **TGFβ**, *collagen 1 A1 (Col1A1)* and *prostaglandin D receptor 2 (PTGDR2)*. We observed AB_AGS_+-driven activation of *TGFβ*; however, whether AB were derived from HCV+ or HCV− cells was not critical for TGFβ expression in HSC (Figure 6A). There were no effects of AB_AGS+_ on ColA1A mRNA levels, while its expression was enhanced by AB_HCV_ and AB_HCV+AGS+_ (Figure 6B). Remarkably, expression of PTGDR2 was up-regulated by AB_AGS_+ by 30% and by AB_HCV+_ -by 2-fold, while AB_HCV+AGS+_ increased the receptor expression by 3.6-fold (Figure 6C). Consistent with AB-mediated activation of inflammasome, we observed no additional effects of HSC pre-treatment with ethanol on induction of pro-fibrotic changes in HSC (not shown).

## 4. Discussion

Hepatocyte injury leads to significant liver dysfunctions. It is known that the chronic hepatocyte damage results in inflammation, myofibroblast activation and progression of liver injury from hepatitis with fibrosis to cirrhosis and even to hepatocellular carcinoma (HCC). Apoptotic hepatocytes engulfed by non-parenchymal cells, Mph and HSC, trigger liver damage progression [15]. There is a high incidence of liver cirrhosis and HCC in HCV-infected people, which is further exacerbated by alcohol abuse [3,16,17]. 

Previously, we demonstrated that alcohol metabolism and particularly, acetaldehyde induces apoptosis of HCV-infected hepatocytes, which promotes virus spread to intact hepatocytes via these AB to generate clustering HCV-infection in the liver [9,18]. The goal of the current study was to test whether these AB contribute to inflammation and fibrosis development by activating liver Mph and HSC. To mimic pro-apoptotic effects of acetaldehyde and HCV and generate AB, we exposed Huh7.5-CYP2E1 (RLW) cells to UV light. RLW cells are able to metabolize ethanol only by CYP2E1, but not ADH and thus, to recapitulate conditions of continuous ADH-induced acetaldehyde production and exposure to this ethanol metabolite, RLW cells were incubated with an AGS described in detail in our previous studies [9,10,11]. These cells/treatments mimic the effects of ethanol on primary human hepatocytes [19]. To test whether HCV antigens or ethanol-metabolism-induced lipid peroxidation adducts expressed in undergoing apoptosis hepatocytes are responsible for inflammatory or pro-fibrotic effects from AB internalization by MDMs or HSC, RLW cells were infected with HCV (JFH1) and/or treated with AGS prior to UV exposure. In addition, alcohol metabolism can change the functions/internalization capacities of those non-parenchymal cells that express alcohol-metabolizing enzymes. To account for this, we used ethanol-pretreated or untreated with alcohol MDMs and HSC that were further exposed to AB_AGS_ to differentiate the effects of adduct-containing AB and ethanol metabolism on inflammatory and pro-fibrotic changes in activated non-parenchymal cells.

As reported before, engulfment of apoptotic hepatocytes by Kupffer cells promoted inflammation in the liver, and these effects were attenuated by gadolinium chloride treatment [5]. We found that engulfment of AB made from HCV-infected RLW cells (an experimental prototype of hepatocytes) by MDMs activate inflammasome in these cells by enhancing expression of *NLRP3, caspase-1*, *IL-1β*, *IL-18, IL-6, TNFα and IL-8.* As already demonstrated, both HCV core and non- structural protein 3 (NS3) proteins can activate inflammasomes [20,21,22]. However, for the first time, we have shown that the source of inflammasome activation in macrophages might be AB generated from parenchymal liver cells. The effects of HCV-containing AB on inflammasomes were attenuated by exposure of RLW cells to AGS. This may be related to the inhibition of HCV replication by lipid peroxidation [23,24], which we confirmed by measuring 4-HNE adducted proteins in AB from AGS-exposed RLW cells. In fact, in AB from the same AGS-pre-treated HCV+ cells, we observed a lower level of HCV core protein, indicating that increased 4-HNE levels corresponded to lower amount of virus in apoptotic RLW cells. While AGS exposure to HCV-infected hepatic cells lowered the magnitude of inflammasome induction in Mph, the pattern of response to AGS-pre-treated AB was similar to AGS-untreated AB, indicating that lipid peroxidation products and specifically, 4-HNE by themselves do not trigger inflammasome activation in MDMs after AB engulfment. Thus, inflammasome induction occurs mainly because of the virus expressed in AB_HCV+RLW_. Interestingly, apoptosis of infected hepatocytes was always considered as a protective mechanism leading to infected cell elimination. However, HCV-containing AB may promote liver inflammation upon engulfment by macrophages. Furthermore, exposure of MDMs to ethanol prior to AB engulfment enhanced activation of inflammasomes by AB, with the highest effects of AB_HCV+_. These effects of ethanol pre-treatment can be attributed to ethanol metabolism, since macrophages express ethanol-metabolizing enzymes, CYP2E1 and ADH, corroborating previous reports [25,26]. When exposed to ethanol, macrophages generate the metabolites (presumably, acetaldehyde and reactive oxygen species (ROS)), which seem to further enhance the AB (and especially, AB_HCV+_)-mediated inflammasome induction. Interestingly, there was no stabilization of CYP2E1 in ethanol-treated MDM, since the level of oxidative stress generated in MDM was probably not enough to suppress proteasome activity that uses CYP2E1 as a substrate for degradation [27,28]. Thus, the ability of macrophages to generate oxidative stress in response to ethanol is lower than in ethanol-treated hepatocytes with CYP2E1 stabilization due to suppressed proteasome activity [29,30,31]. This suggests that various levels of oxidative stress differentially regulate both proteasome activity and the expression of ethanol-metabolizing enzymes in HCV-infection combined with alcohol exposure [32]. We should also acknowledge the possibility that alcohol metabolism affects the ability of macrophages to phagocytose AB. However, in this study, we did not focus on the effects of ethanol metabolism on phagocytosis of AB by macrophages, which may be reduced depending on experimental conditions [33]. Since phagocytosis is regulated by multiple factors and is model-specific [34], it should be the subject of special studies. Here, we compared the relative ability of AB from infected and uninfected RLW cells to affect inflammasome induction in the presence or absence of ethanol. Thus, even if phagocytosis is suppressed by ethanol, this suppressing effect will equally impact the response of macrophages to AB from infected and uninfected liver cells. In addition, ethanol has been used for macrophage pre-treatment and was removed at exposure of cells to AB. However, the aspect of phagocytosis regulation by ethanol metabolism should be addressed in future studies.

The next question we asked was whether the regulatory effects of hepatocyte AB on inflammasome induction is specific only for macrophages or can it also be determined in other non-parenchymal cells, such as HSC. It has been demonstrated that phagocytosis of AB by LX-2 cells is accompanied by activation of signaling pathways [35,36,37]. We indeed found a slight enhancement of *NLRP3* and about 30% increased *IL-1β*-expression after engulfment of AB from hepatocytes. However, there was no difference in the magnitude of *IL-1β* activation by AB generated from HCV+ or AGS-treated RLW cells, indicating that both stimuli can activate inflammasomes in HSC. In the literature, we neither found data about inflammasome expression in HSC induced by HCV and its combination with ethanol, nor the role of AB in this induction. Furthermore, the highest increase in some pro-inflammatory chemokines as *MCP-1, MIP-2* and *IL-8*-expression levels in HSC were detected when cells were exposed to AB_AGS+RLW_ cells expressing the lipid peroxidation marker, 4-HNE-adducted proteins, while the influence of AB_HCV+_ was minimal. No additional effects of ethanol pretreatment have been observed in these cells on AB-induced inflammasome activation and pro-inflammatory cytokine expression. In HSC cells, in addition to inflammasomes, we measured the expression of pro-fibrotic markers, Col1A1 and PTGDR2 in response to AB engulfment. Remarkably, Col1A1 expression was mainly induced by AB_HCV+,_ with no additional effect coming from AB_AGS_, while we observed an additive effect of AB_HCV+ AGS_ on PTGDR2, suggesting that acetaldehyde-induced adducts (presumably, 4-HNE) expressed in undergoing apoptosis HCV+ hepatocytes may stimulate the fibrotic potential of HSC. Since ethanol exposure to MDMs significantly up-regulated an important HSC activator, *TGFβ* after AB engulfment, we would assume that ethanol-pretreated macrophages will significantly contribute to HSC-induced pro-fibrotic changes triggered by TGFβ release. Our data are in line with already published pro-fibrotic effects of AB engulfment by HSC [38] and pro-fibrotic activation of HSC with AB from Con1-bearing (HCV genotype 1) Huh cells that express only non-structural HCV proteins [7]. However, we infected Huh7.5 cells with full-genome JFH1 (genotype 2a) virus to generate AB for our study, which has been also done in a context of mimicking the effects of ethanol metabolism on liver cells. In our hands, in macrophages, the decreased response to acetaldehyde-exposed (AGS-treated) AB was accompanied by the reduction in HCV core protein levels due to elevated lipid peroxidation products, which may suppress viral replication. It appears that in HSC, lipid peroxidation and concomitant increase in 4-HNE-protein adducts in AB even enhanced the pro-fibrotic effects of HCV. 

## 5. Conclusions

**Figure 7:**
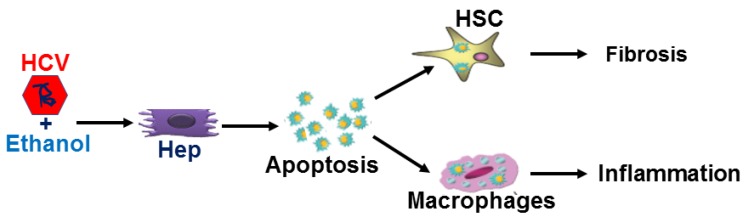
The possible mechanisms of how HCV and ethanol metabolism promote liver inflammation and fibrosis. Hepatocytes: Hep.

In conclusion, our findings strongly suggest that when apoptosis is induced in HCV-infected hepatocytes (Hep) by ethanol metabolism, virus/viral proteins as well as lipid peroxidation-adducted proteins packaged in AB may serve as a vehicle for delivery of parenchymal cell cargo to non-parenchymal cells, macrophages and hepatic stellate cells, to activate inflammasome and pro-fibrotic genes and promote liver inflammation and fibrosis (Figure 7).
